# The Evaluation of the Clinical Effectiveness and Safety of Biologic Therapies in Psoriasis–Real-World Evidence up to 64 Weeks of Therapy

**DOI:** 10.3390/medsci14040502

**Published:** 2026-08-20

**Authors:** Patrycja Lemiesz, Julia Nowowiejska-Purpurowicz, Tomasz W. Kaminski, Iwona Flisiak

**Affiliations:** 1 Department of Dermatology and Venereology, Medical University of Bialystok, Zurawia 14 St., 15-540 Bialystok, Poland; julia.nowowiejska@umb.edu.pl (J.N.-P.); iwona.flisiak@umb.edu.pl (I.F.); 2 Department of Medicine, Pittsburgh Heart, Lung and Blood Vascular Medicine Institute, University of Pittsburgh, Pittsburgh, PA 15260, USA; tkaminski@versiti.org; 3 Thrombosis and Hemostasis Program, Versiti Blood Research Institute and Blood Center of Wisconsin, Milwaukee, WI 53226, USA

**Keywords:** psoriasis, biological drugs, TNFα inhibitors, risankisumab, guselkumab, adalimumab

## Abstract

**Background**: Biological treatment of psoriasis has revolutionized the therapy of this disease, in most cases enabling complete remission of skin lesions. **Methods**: This study aimed to analyze real-world evidence from 165 patients (mean age 38.1 ± 5.66) treated with 11 biological drugs at our Dermatology Department. **Results**: Skin lesion severity, expressed by Psoriasis Area and Severity Index (PASI) and Body Surface Area (BSA), improved significantly throughout the treatment, along with the quality of life, expressed by Dermatology Life Quality Index (DLQI) (all *p* < 0.0001). We observed a statistically significantly higher reduction in skin lesion severity and life quality improvement with IL-23 and IL-17 inhibitors compared to TNFα inhibitors (both *p* < 0.0001). Secondary loss of response was observed in 26.7% of patients treated with anti-TNF agents, compared to 15.0% in the anti-IL-23 group and 8.3% in the anti-IL-17 group (χ^2^ test, *p* = 0.0683). Patients who had been treated in the past with biologics had significantly higher PASI and BSA at week 16 of therapy compared to those who were bio-naïve. A positive correlation was demonstrated between PASI at 16 weeks and BMI (R = 0.15; *p* = 0.0396). There was no correlation between the duration of psoriasis, sex, or age of patients when introducing biologics and PASI at any of the timepoints (*p* > 0.05). The most frequent side effects were infections, occurring mainly in patients on anti-TNFα agents and bimekizumab. **Conclusions**: IL-17 and IL-23 inhibitors demonstrated superior efficacy compared to TNFα inhibitors, with higher rates of complete skin clearance and a lower tendency toward loss of response. The worst efficacy and the highest rate of loss of response were observed in case of adalimumab. These findings support the increasing role of IL-17 and IL-23 inhibitors as preferred therapeutic options in psoriasis. Previous biologic exposure and higher BMI seem to be associated with poorer therapeutic outcomes. Overall, biological treatment is an effective, rapid, and safe therapeutic option.

## 1. Introduction

Psoriasis is a chronic and incurable skin disease that affects a significant number of patients worldwide. It is associated with many comorbidities, particularly psoriatic arthritis (PsA) and metabolic syndrome, which lead to decreased life expectancy and lower quality of life [1,2,3,4,5].

Treatment of psoriasis has evolved over the years. The oldest antipsoriatic agents were applied topically and were coal-tar or dithranol derivatives [6]. Topical steroids, calcineurin inhibitors, and vitamin D derivatives have also been widely used [7]. As for systemic agents, the first therapeutics were methotrexate, acitretin, and cyclosporin A, which are widely available. Psoralen ultraviolet A (PUVA) has also appeared, followed by narrowband UVB therapy [6]. In some countries, apremilast and dimethyl fumarate are also available. All mentioned methods have various efficacies, do not usually enable complete clearance of skin lesions, and are characterized by different extents of side effects [8].

Fortunately, several decades ago, patients with psoriasis gained access to biological treatment, which was associated with better efficacy, along with fewer adverse effects [9]. The oldest group of biological agents is TNFα inhibitors; later, IL-17 inhibitors appeared, and a few years ago, IL-23 inhibitors.

This reflects the pathogenesis of psoriasis. The IL-23/Th17 axis is considered a central pathway in psoriasis, as IL-23 is placed at its beginning and promotes Th17 and Th22 cells, which further secrete IL-17 and IL-22. Both cytokines are known to stimulate epidermal proliferation, which is a hallmark of psoriasis. TNFα is another molecule important in the pathogenesis of this disease, being secreted by the Th1 cell line upon stimulation by IL-12 [10]. IL-23 is composed of two subunits (p19 and p40), with the p40 subunit also being a component of IL-12. This explains the function of risankizumab, guselkumab, and tildrakizumab, which target only p19, whereas an older drug, ustekinumab, targets both p19 and p40 [11]. Regarding IL-17, it occurs in six isoforms named A to F. Notably, IL-17A is characterized by the highest biological activity in general and occurs as a homodimer or a heterodimer with IL-17F. On the other hand, IL-17F is thought to be more abundantly expressed in psoriatic tissue [12]. Secukinumab and ixekizumab inhibit IL-17A selectively, whereas bimekizumab inhibits both IL-17A and F. Regarding TNFα inhibitors, the first drug from this class was etanercept, which is different from the rest, since it is a fusion protein and acts as a receptor for TNFα. The other four drugs (infliximab, certolizumab pegol, golimumab, and adalimumab) are monoclonal antibodies, which bind to the TNFα itself and prevent it from further binding to its receptor [13].

Although there is extensive data from clinical trials evaluating the efficacy and adverse effects of antipsoriatic drugs, as well as access to data from head-to-head studies comparing particular agents with each other, these patients are typically selected and do not accurately reflect the general population. Therefore, studies analyzing data from real-world clinical practice are necessary.

This study aimed to analyze real-world evidence from patients treated with biological drugs at our Department of Dermatology. The specific objective was to compare the efficacy of particular drugs with each other, analyze their side effects, and assess whether specific demographic or clinical patient features, as well as previous treatment (both classic and biological), affect the response to biological agents. Such an analysis adds to the current state of knowledge and allows for treatment personalization tailored to particular patients.

## 2. Materials and Methods

The approval for the presented study was provided by the Bioethics Committee at the Medical University of Bialystok, Białystok, Poland (APK.002.236.2025, date of approval: 22 May 2025). Due to the retrospective nature of the study, which involved the analysis of collected and anonymized patient data, it was exempt from the requirement to obtain informed consent from patients. The study was conducted according to the Helsinki Declaration; the collection and processing of data were carried out in accordance with applicable law, with a focus on protecting patients, particularly their privacy. In our country, patients are treated with biological agents mainly in the National Health Fund-funded program; hence, such individuals were enrolled in this analysis. Currently, there are 11 biological agents available in our country (infliximab, adalimumab, etanercept, certolizumab pegol, ustekinumab, secukinumab, ixekizumab, bimekizumab, guselkumab, tildrakizumab, and risankizumab).

### 2.1. Patients

Patients treated due to plaque psoriasis with biological agents in the Department of Dermatology and Venereology, Medical University and Teaching Hospital of Bialystok, due to plaque psoriasis were included in this study. All patients were Caucasians. No age or sex restrictions were determined.

### 2.2. Data Collection

Medical records from 2012 were screened by two doctors (PL, JNP). The following data were extracted: demographic information (age, sex), accompanying diseases (abnormal body weight, smoking, alcohol abuse), medical history (family history of the disease, disease duration, previous treatment and its efficacy), and information on the current therapy (chosen drug, treatment duration, its efficacy, side effects). Treatment failure was defined as primary when the patient did not meet the criteria of the response to therapy at week 16. Secondary treatment failure was analyzed in two ways: mild secondary failure, when the patient reached PASI 100, but later at some point, mild lesions recurred; and total secondary failure, when the patient achieved PASI 100, but later at some point, the lesions recurred to the extent at least as high as initially.

Patients were monitored throughout the treatment—before the initiation and after 16 weeks; subsequently, the monitoring took place every 24 weeks (6 months). Considering the above, we enrolled only patients who were monitored for at least 16 weeks. For 64.4% of subjects, we obtained data up to 10 months, and for 46.7% up to 16 months of therapy. During follow-up appointments, the following aspects are evaluated: severity of skin lesions (by psoriasis area and severity index—PASI, and body surface area—BSA), quality of life (by dermatology life quality index—DLQI), and basic laboratory parameters.

### 2.3. Statistical Analysis

Statistical analyses were performed using GraphPad Prism 11 (GraphPad Software, San Diego, CA, USA). Continuous variables were presented as the mean ± standard error of the mean (SEM) or as the median with the interquartile or full range (IQR), depending on data distribution. Categorical variables were expressed as numbers and percentages.

Normality of data distribution was assessed using the Shapiro–Wilk test. Comparisons between two groups were performed using the unpaired Student *t*-test or Mann–Whitney U test, as appropriate. Paired comparisons between baseline and follow-up visits were analyzed using the paired Student *t*-test or Wilcoxon signed-rank test. Comparisons among multiple groups were performed using one-way ANOVA with appropriate post hoc multiple-comparison correction or the Kruskal–Wallis test, depending on data distribution.

Changes in PASI, BSA, and DLQI during treatment were analyzed at predefined follow-up time points (week 16, week 40, and week 64). Analyses at each follow-up time point were based on available cases only; patients without data at a given visit were excluded from the corresponding analysis, and no missing-data imputation was performed. Correlations between selected clinical and demographic parameters were assessed using Spearman’s rank correlation coefficient.

Categorical variables were compared using the chi-square test or Fisher’s exact test, as appropriate.

All statistical tests were two-sided, and a *p* value < 0.05 was considered statistically significant.

## 3. Results

### 3.1. Basic Characteristics

A total of 165 patients who completed at least 16 weeks of biological therapy were enrolled in the study. The basic information about the study populations is presented in Table 1.

### 3.2. Applied Treatment

**Figure 1:**
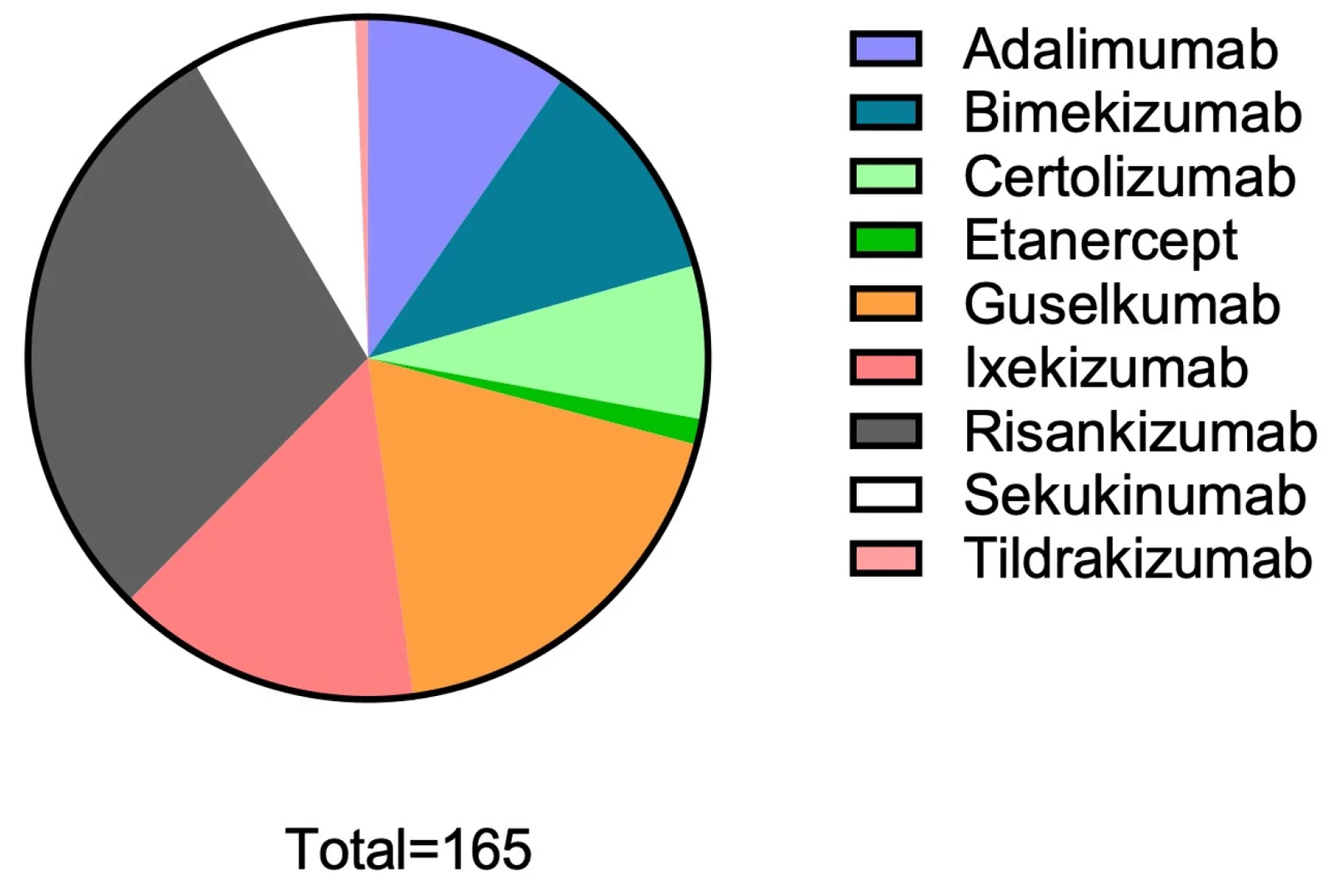
Diagram illustrating the number of patients treated with particular agents.

In our cohort, patients received the following agents: 48 patients were administered risankizumab, 31 guselkumab, 24 ixekizumab, 18 bimekizumab, 16 adalimumab, 13 secukinumab, 12 certolizumab pegol, 2 were given etanercept, and 1 was administered tildrakizumab (Figure 1).

Seven patients required a change in drug, usually from adalimumab, either due to lack of primary efficacy or eczematoid eruptions. Four patients required an increase in the dosage (if possible, based on the product characteristic, mainly certolizumab).

Baseline characteristics of patients after division into particular drug classes are presented in Table 2.

There were no statistically significant differences between the patients treated with particular drug classes in terms of their BMI or addictions (*p* > 0.05). Patients treated with IL-23 inhibitors had initially more severe (higher PASI and BSI) and long-lasting psoriasis compared to those treated with IL-17 inhibitors (*p* < 0.01) (Table 2).

### 3.3. Treatment Effectiveness

**Figure 2:**
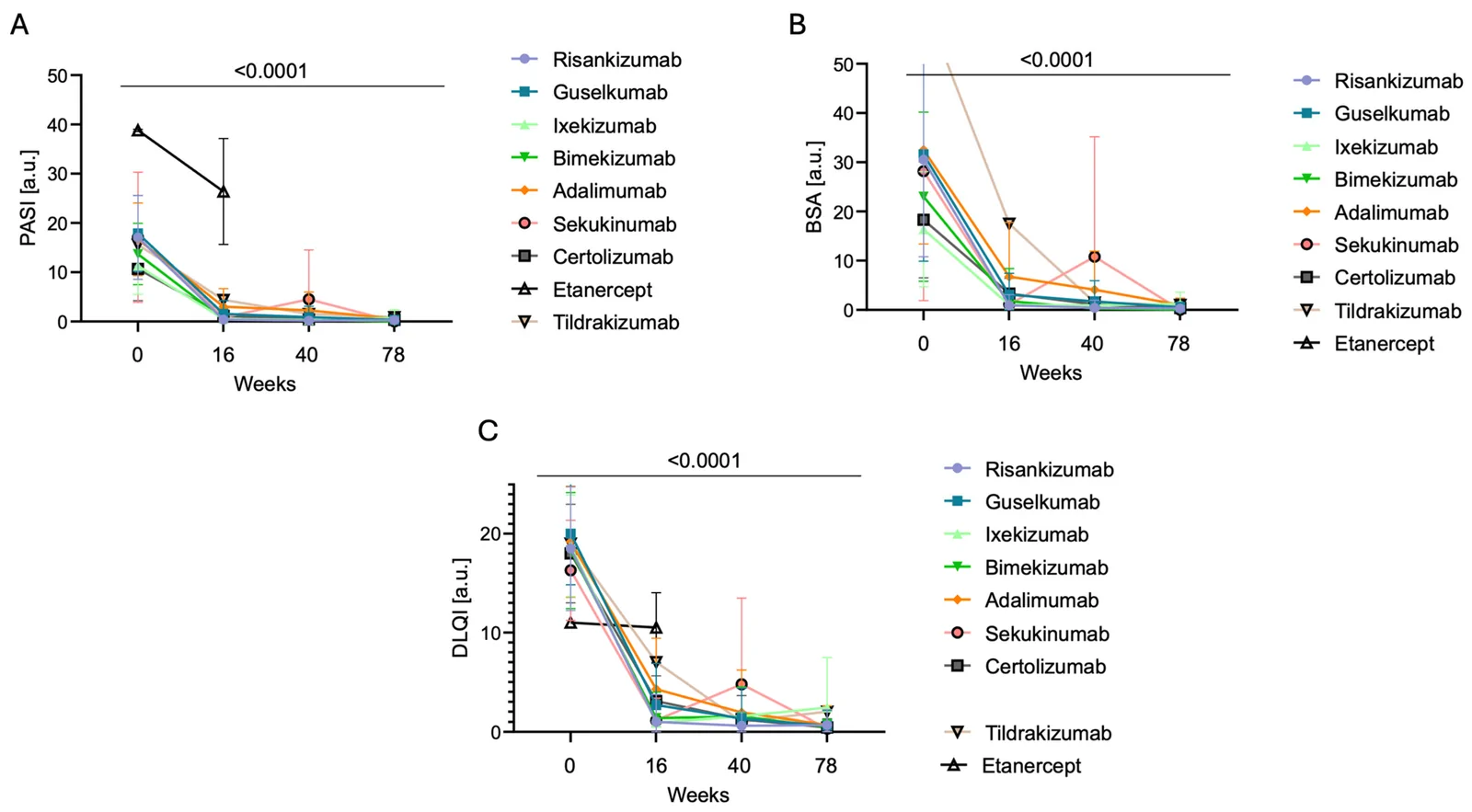
Reduction in skin lesion severity assessed by PASI (**A**) and BSA (**B**), as well as quality of life assessed by DLQI (**C**), throughout the therapy with each of the drugs. PASI, psoriasis area and severity index; BSA, body surface area; DLQI, dermatology life quality index.

Skin lesion severity expressed by PASI and BSA improved significantly throughout the treatment, along with the quality of life, expressed by DLQI (all *p* < 0.0001) (Figure 2A–C).

**Figure 3:**
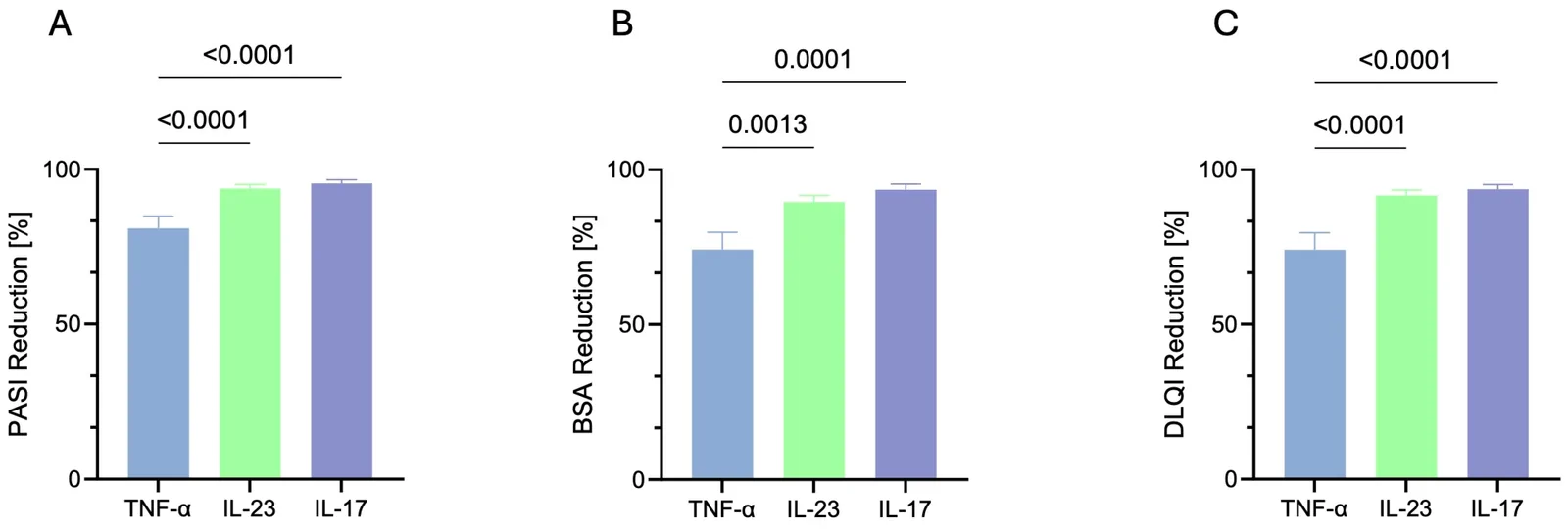
Reduction in skin lesion severity assessed by PASI (**A**) and BSA (**B**), as well as quality of life assessed by DLQI (**C**), for patients on each class of drugs separately. PASI, psoriasis area and severity index; BSA, body surface area; DLQI, dermatology life quality index.

After the division of the drugs into three main classes, we observed a statistically significantly higher reduction in skin lesion severity and life quality improvement with IL-23 and IL-17 inhibitors compared to TNFα inhibitors (both *p* < 0.0001) (Figure 3A–C).

#### 3.3.1. Sixteenth Week of Therapy

The median PASI after 16 weeks of therapy was 0 (0–13.2), the median BSA was 0 (0–28), and the median DLQI was 0 (0–18). All differences were statistically significant compared to scores before the treatment (*p* < 0.0001). Overall, 98.8% patients achieved PASI50, 75.75% achieved PASI90, and 56.4% achieved PASI100; 4.2% of patients required a change in drug due to inefficacy or adverse events.

At week 16, PASI90 was achieved by 43.3% of patients receiving TNF-α inhibitors, compared with 80.0% and 81.3% of those receiving IL-17 and IL-23 inhibitors, respectively. PASI100 was achieved by 23.3%, 63.6%, and 58.8% of patients, respectively. In contrast, PASI50 response rates were high across all three treatment classes (93.3%, 100%, and 98.8%, respectively). The median time to achieve PASI100 was longer in the TNF-α inhibitor group than in the IL-17 and IL-23 inhibitor groups (10, 4, and 3 months, respectively).

#### 3.3.2. Fortieth Week of Therapy

The median PASI after 40 weeks (10 months) of therapy was 0 (0–13.2), the median BSA was 0 (0–28), and the median DLQI was 0 (0–18). All differences were statistically significant compared to scores before the treatment (*p* < 0.0001). Overall, 97.2% of patients achieved PASI50, 85.8% achieved PASI 90, and 60.3% achieved PASI 100. None of the patients required a change in drug.

#### 3.3.3. Sixty-Fourth Week of Therapy

The median PASI after 64 weeks (16 months) of therapy was 0 (0–6.4), the median BSA was 0 (0–8), and the median DLQI was 0 (0–18). All differences were statistically significant compared to scores before the treatment (*p* < 0.0001). Overall, 98.7% of patients achieved PASI50 and PASI 90, and 77.9% achieved PASI 100. None of the patients required a change in drug.

### 3.4. Additional Observations

Overall, 74.5% of patients were able to achieve PASI 100 at any time during the 16 months of observation. The median time to achieve PASI 100 in those who did so was 4 months (IQR 1–4). This period was shortest for risankizumab and longest for adalimumab.

Primary non-response (therapy failure) was observed in five individuals in week 16, who were mainly treated with adalimumab and etanercept, leading to a change in the drug. The loss of response to treatment (secondary failure) was analyzed in two ways. Firstly, the proportion of patients in whom any mild lesions reoccurred at some point after they had initially achieved PASI 100 was 11.5%. This loss was observed after a mean of 13.3 ± 1.28 months, mainly in case of guselkumab and adalimumab. Secondly, we assessed total secondary failure, meaning the proportion of patients in whom the lesions reoccurred with severity as high as before the treatment, which was 2.4%. This loss was observed after a mean of 15.8 ± 4.25 months, mainly in the case of adalimumab. Secondary loss of response (combined mild and complete) was observed in 26.7% of patients treated with anti-TNF agents, compared to 15.0% in the anti-IL-23 group and 8.3% in the anti-IL-17 group (χ^2^ test, *p* = 0.0683).

Small residual lesions remaining constantly in the same place despite the resolution of other lesions were observed in 8.4% patients. Only one was biopsied to exclude cutaneous lymphoma due to the clinical and dermoscopic picture.

### 3.5. Adverse Events

Overall, 4.8% patients reported more frequently recurring bacterial or viral infections compared to the time before the therapy (most commonly in subjects treated with adalimumab and certolizumab pegol), while 3% reported fungal infections (mainly on bimekizumab). Malignant neoplasm was diagnosed only in one patient (prostate cancer) at the beginning of therapy. None of the patients experienced inflammatory bowel diseases or reactivation of tuberculosis. Two patients treated with adalimumab experienced eczematoid lesions.

### 3.6. Analysis of Laboratory Parameters

We compared basic laboratory parameters, particularly inflammatory parameters, before and after 16 weeks of treatment. There were no statistically significant differences between the values before and after; however, the concentration of CRP tended to decrease, whereas WBC and PLT remained stable (Table 3).

### 3.7. Analysis of Specific Locations–High-Impact Areas

**Figure 4:**
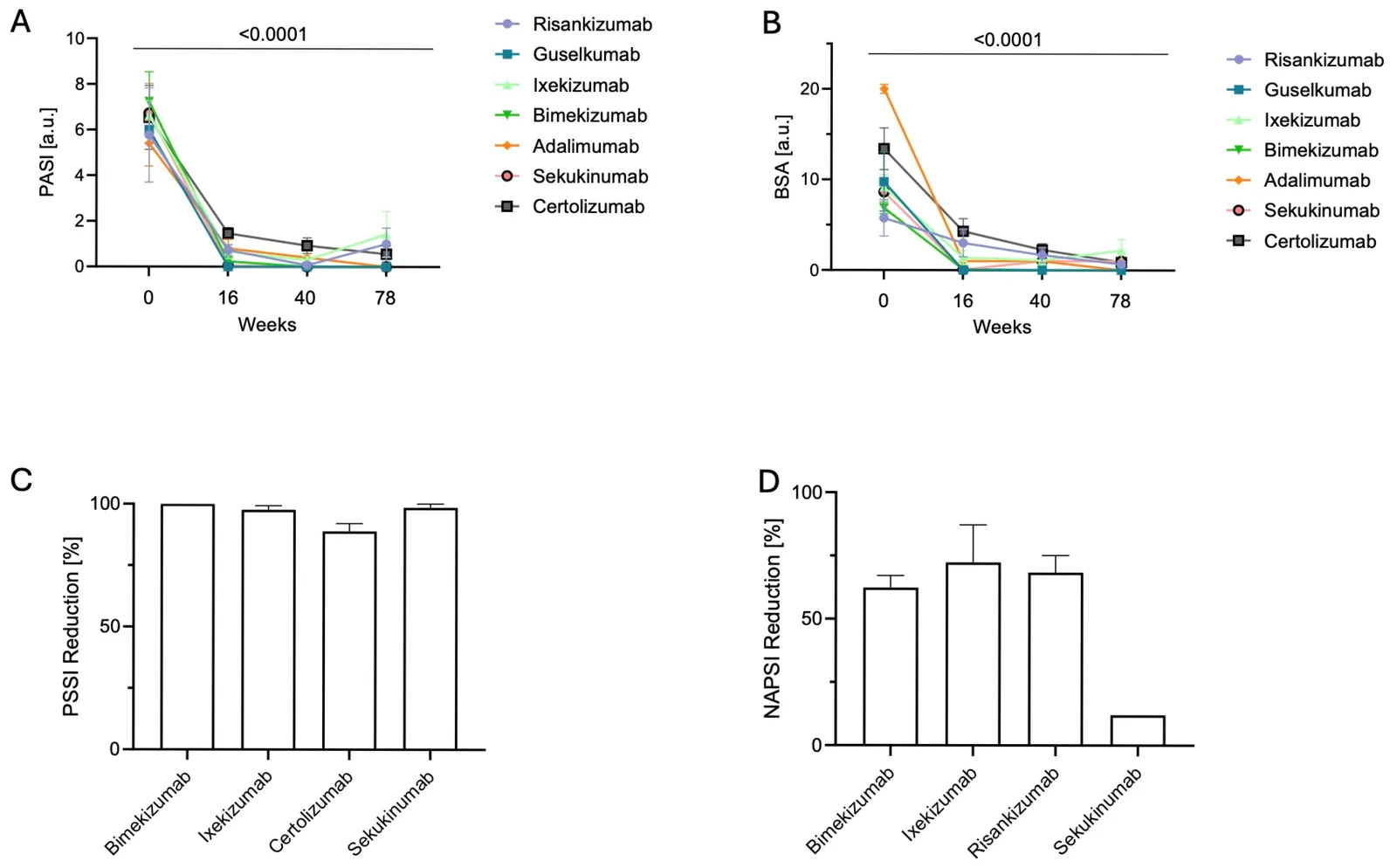
Comparison of skin lesion reduction between particular drugs in high-impact areas: (**A**) reduction in PASI in all affected patients; (**B**) reduction in BSA in all affected patients; (**C**) reduction in NAPSI in patients with nail involvement; (**D**) reduction in PSSI in patients with scalp involvement. PASI, psoriasis area and severity index; BSA, body surface area; dermatology life quality index; NAPSI, nail apparatus psoriasis severity index; PSSI, psoriasis scalp severity index.

Overall, 94.5% of patients experienced the involvement of specific locations. The most frequently involved area was the scalp (82.4%), followed by the nail unit (50.9%). Some patients received biological drugs due to the high impact of involved areas, whereas the total severity of lesions as expressed by PASI was not very high. When comparing the reduction in PASI and BSA of patients with high-impact areas of involvement (Figure 4A,B), as well as PSSI (in cases of scalp involvement) and NAPSI (in cases of nail involvement) (Figure 4C,D), there were no statistically significant differences between particular drugs (*p* > 0.05).

### 3.8. Influence of Previous Therapy on the Response to Current Treatment

**Figure 5:**
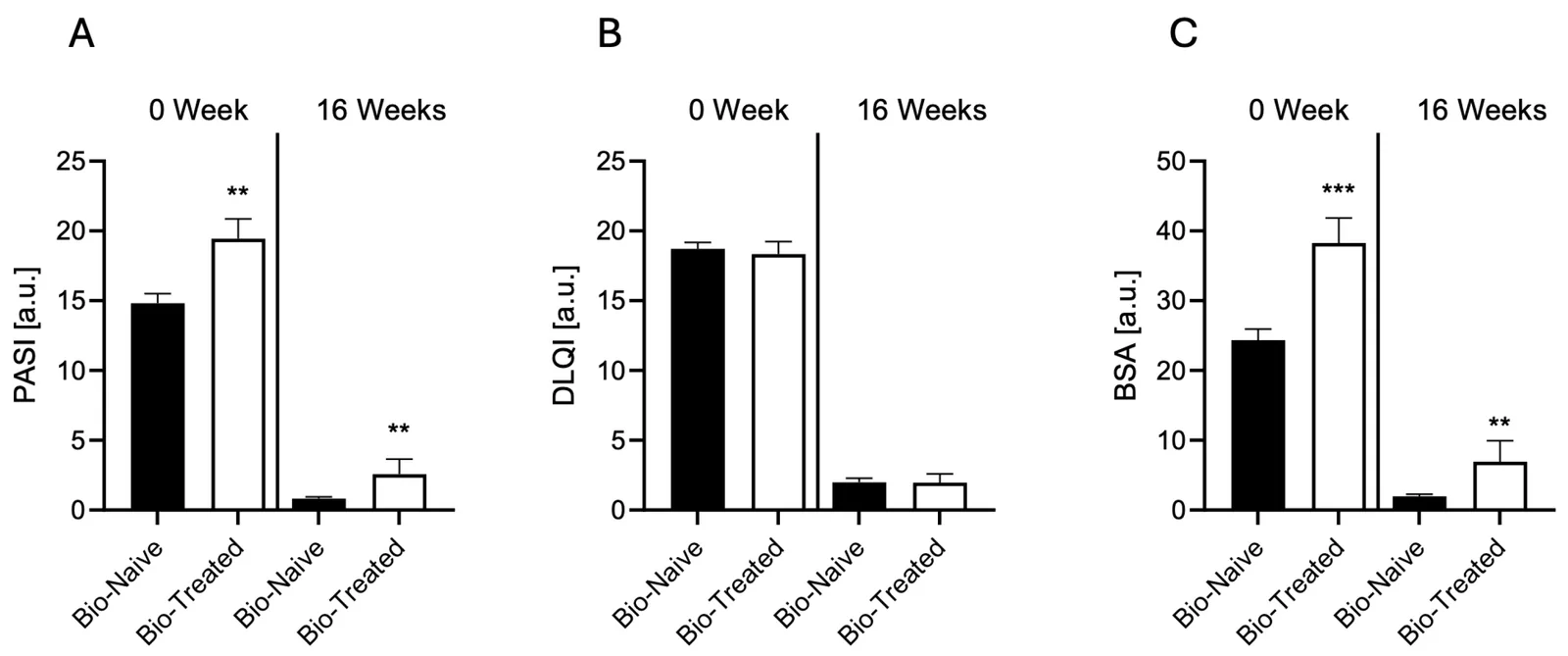
The comparison of PASI (**A**), DLQI (**B**) and BSA (**C**) between patients previously treated and not previously treated (bio-naïve) with biologics. **/*** indicates statistically significant differences with *p* < 0.01/0.0001, respectively; PASI, psoriasis area and severity index; BSA, body surface area; DLQI, dermatology life quality index.

Overall, 90% patients had previously been treated with systemic agents: 76.3% with methotrexate, 63.6% with acitretin, 33.9% with cyclosporin A, 2.4% with PUVA, and 22.4% with biological agents. Patients who had been treated in the past with biologics had significantly higher PASI and BSA at week 16 of therapy compared to those who had not been treated with these agents (bio-naïve) (Figure 5A–C).

### 3.9. Influence of Demographic and Clinical Patients’ Features on the Response to Current Treatment

Spearman’s rank correlation analysis was conducted between the PASI score at 16 weeks of treatment and selected clinical and demographic parameters. A statistically significant positive, albeit weak, correlation was demonstrated between PASI at 16 weeks and BMI (R = 0.15; 95% CI: 0.0031–0.30; *p* = 0.0396). We also compared the clinical response to therapy (PASI, BSA) in week 16 in patients treated with particular drug classes who had normal and abnormal body weight (overweight/obesity); however, there were no significant differences between the drug subtypes (*p* > 0.05).

There was no correlation between the duration of psoriasis, sex, or the age of patients when introducing biologics and PASI in any of the time points (*p* > 0.05).

## 4. Discussion

Biological treatment of psoriasis has revolutionized the therapy of this disease, in most cases enabling complete remission of skin lesions. Thanks to these drugs, patients’ quality of life has improved, and many of them have been able to return to their professions and social activities. Although we possess data from clinical trials, these are highly selected populations; hence, information from the real world is of great interest. To the best of our knowledge, this is one of the few analyses of biological agents in children and adults with psoriasis [14,15].

Approximately 53% of our patients reported a positive family history of psoriasis. According to the recent literature, we observed a relatively higher proportion of patients with a positive family history of psoriasis, which in other observational studies ranged from 10.8% to 47% [16,17,18]. Certainly, psoriasis is characterized by a genetic predisposition, and currently, there are over 60 chromosomal loci identified that increase susceptibility [19]. PsA was diagnosed by a rheumatologist in 29% of all patients, which is consistent with available data estimating the risk of arthritis to be up to 30% [20].

Regarding the clinical manifestation of psoriasis, over 80% of patients had scalp lesions, and over 50% of them displayed nail involvement, which adds to the epidemiological data. In our study, we did not manage to prove a superior efficacy of any of the drugs in the high-impact areas. This suggests that all investigated biologic classes may provide satisfactory control of psoriasis in difficult anatomical locations, although larger comparative studies are needed. Only a few studies have compared the efficacy of biologics in high-impact areas head-to-head. In a recent paper by Yan et al., IL-17A inhibitors were overall more effective in nail lesion therapy than IL-23 inhibitors [21], which was indirectly supported by previous studies showing the superiority of ixekizumab [22,23]. Regarding scalp involvement, IL-17 and 23 inhibitors were more effective overall than TNFα inhibitors [24,25].

In our cohort, we were able to prove the superior efficacy of IL-23 and IL-17 inhibitors over TNFα inhibitors in skin lesion reduction and quality of life improvement. Studies comparing the effectiveness of biological agents head-to-head are limited, despite this information being crucial for clinicians to decide on the choice of a particular agent. The existing data on direct comparisons between the drugs available mainly focus on ustekinumab vs. etanercept, secukinumab, ixekizumab, brodalumab, and risankizumab [26,27,28,29,30]. Secukinumab has been compared to risankizumab and bimekizumab [12,31], whereas guselkumab has been compared to ixekizumab and adalimumab [32,33]. Overall, TNFα inhibitors were characterized by lower efficacy than IL-17 and IL-23 inhibitors, and IL-17 inhibitors were found to have lower efficacy than IL-23 inhibitors, which is partially reflected by our study. Notably, patients treated with IL-23 inhibitors had more severe psoriasis initially compared to other classes, yet they still achieved great clinical improvement.

Demographic factors are also frequently discussed as potentially influencing the course of treatment. In our study, only higher BMI seemed to be associated with poorer therapeutic outcomes, whereas before therapy initiation, there were no significant differences in BMI of patients who received particular drug classes. Age, sex, and addictions were not associated with clinical outcomes. This is partially consistent with the literature, since obesity has been proven to influence the efficacy of both biological and conventional drugs, as well as drug survival [34]. We also compared the clinical response to therapy in patients treated with particular drug classes who had a normal and abnormal body weight; however, there were no advantages of any class in terms of skin lesion reduction in obese individuals.

One of the main aims of this analysis was to thoroughly review previous psoriasis therapy and its influence on the current biological treatment. Over 90% of patients had previously been treated with at least one systemic agent. This is due to several factors. Firstly, access to biological treatment is limited in our country, and patients must demonstrate the ineffectiveness of previous treatment, adverse effects, or contraindications to two classic methods of systemic treatment. In the case of children, biological treatment may be implemented after ineffective topical therapy. Only 22.4% of all patients had previously been treated with at least one biological agent (in our country, in clinical trials), so approximately 78% were bio-naïve. Our analysis revealed that subjects who had not previously been treated with biological agents experienced a more significant improvement in the severity and area of their skin lesions. Similar observations were made in recent studies by di Giulio et al. and Hjort et al. [35,36]. The reasons for a change in drug may vary and are not always reported in scientific papers; hence, it is difficult to assess the characteristics of patients who required such a change [35]. Nevertheless, it could be explained by several reasons. Patients who are bio-experienced are a highly selected population. Patients in whom biological treatment is administered usually suffer initially from severe forms of psoriasis and have probably become resistant to other forms of therapy. Their psoriasis duration is longer, and they may have more comorbidities. Another explanation is their previous exposure to biological agents and the secretion of anti-drug antibodies, which sometimes may have the ability to diminish the drug’s efficacy [37]. Moreover, it has been reported that patients with primary treatment failure are prone to a worse clinical response to another drug than those with secondary failure [35]. In addition to these hypotheses, certain genetic factors must be taken into account when analyzing the response to therapy, and an increasing number of publications are appearing on this topic [38,39]. Our observations remain clinically relevant in the era of multiple biologic switches and support the concept that earlier introduction of highly effective agents may improve long-term outcomes.

The median psoriasis duration before the introduction of biological therapy in our patients was over 16.7 years, which reflects the limited availability of these drugs in our country, but it did not correlate with PASI at any of the timepoints. The GUIDE study by Schäkel et al. suggested that the shorter duration of psoriasis (<2 years) correlates with a so-called super-response and indicated that the earlier we introduce biological therapy, the better results we obtain [40]. Unfortunately, we were not able to observe this phenomenon in our cohort, since the majority of patients received biological treatment only after several years of disease duration, making comparison between the groups difficult. Nevertheless, patients on IL-23 inhibitors, despite having the longest disease duration, presented favorable outcomes compared to TNFα inhibitors.

Primary and total secondary treatment failure was observed mainly in subjects treated with adalimumab, whereas mild secondary treatment failure was observed in patients on guselkumab. In an Australian study, the lowest drug survival, defined as the time from initiation to discontinuation, was demonstrated for etanercept and adalimumab [41], which is consistent with our observation. The highest survival rate was reported for guselkumab and ixekizumab (risankizumab not reported) [41], but in another paper analyzing all IL-17/23 inhibitors, it was observed for risankizumab [10]. In our cohort, we never discontinued therapy with guselkumab, risankizumab, or ixekizumab, because we never encountered primary or total secondary failure; however, we observed mild secondary failure in several subjects on guselkumab, usually between the first and second year after initiation. Secondary treatment failure (combined mild and total) was observed mainly in patients treated with anti-TNFα agents, compared to the anti-IL-23 and anti-17 groups. Although not statistically significant, a clear numerical trend toward a higher rate of loss of response was observed in the anti-TNFα cohort, with more favorable durability in IL-17 and IL-23 inhibitors. Based on the aforementioned head-to-head comparisons, IL-23 inhibitors are characterized by a persistent long-term response [34]. According to the BADBIR registry, it is age that has the most significant impact on biologic agents’ survival, which is related to their class. Younger patients are more likely to discontinue anti-TNFα agents due to their ineffectiveness, whereas older patients are more likely to discontinue a drug of any class due to side effects [42]. Considering that the primary and total secondary failure occurred in our cohort mainly in subjects on TNFα inhibitors, as well as our observations and literature findings about the long survival of IL-23 and IL-17 inhibitors, we conclude that they should be the preferred therapeutic option.

As for adverse events, the highest rate was observed in the TNFα inhibitors group, namely infections and eczematoid reactions. Our findings are consistent with data from the literature, which identify these reactions as one of the common complications of TNFα antagonists, affecting 5–20% of patients treated with drugs in this class [43].

We noted five cases of fungal (*Candida albicans*) infections, which occurred only in patients treated with IL-17 inhibitors, particularly bimekizumab. This is a complication well-documented in the literature of this drug class, with an incidence rate of 1.7% for secukinumab, 3.3% for ixekizumab, and as high as 6–21% among patients treated with bimekizumab [44]. One patient experienced fungal esophagitis twice, which resulted in drug discontinuation. Our opinion is that anti-IL-17 agents, particularly bimekizumab, should be administered only in subjects without additional risk factors, namely young, immunocompetent patients with normal body weight without diabetes mellitus. We noted a significant increase in liver enzyme activity in a few patients treated with guselkumab, which is also reflected in the clinical trials of this drug [45]. We recommend that it not be the primary choice in patients who are obese, have liver disease, or abuse alcohol. We did not observe any side effects during the therapy with risankizumab. Notably, none of the patients experienced tuberculosis reactivation or inflammatory bowel disease induction during the treatment. Malignant neoplasm was diagnosed only in one patient, and it was not related to the biological agent.

Interestingly, several patients developed small residual lesions that persisted in the same anatomical locations despite otherwise complete or near-complete disease control. This phenomenon may reflect local tissue-resident memory (TRM), which has been increasingly implicated in psoriasis recurrence. Persistent TRM T cells have been proposed as one of the mechanisms responsible for relapse in previously affected areas despite systemic suppression of inflammation [46]. There have been studies demonstrating a reduction in psoriasis in TRM cells after biological therapy, specifically regarding guselkumab, while this was not shown in treatment with secukinumab [47]. Of note, residual, persistent lesions always have to be examined clinically and dermoscopically when making a decision about biopsy in order to exclude other skin conditions.

The main limitations of this analysis are its single-center nature, the variability in the number of patients treated with each agent, and the complete lack of patients treated with infliximab and ustekinumab for full comparison. Another limitation could be the various durations of therapy for the analyzed patients after week 16. The unequal duration of follow-up represents an additional limitation, as only 64.4% and 46.7% of patients had available data at weeks 40 and 64, respectively. This may reduce the precision of long-term estimates and introduce potential attrition bias; therefore, longer-term results should be interpreted cautiously. Because treatment allocation was non-randomized, differences in baseline characteristics and other unmeasured factors may have influenced the observed comparative effectiveness between biologic classes; therefore, these findings should be interpreted as real-world associations rather than causal treatment effects.

## 5. Conclusions

Our analysis adds to the current state of knowledge about the biological treatment of psoriasis based on real-world data. Biological therapy demonstrated high effectiveness in the treatment of psoriasis, leading to significant improvements in skin lesion severity and quality of life. IL-17 and IL-23 inhibitors demonstrated superior efficacy compared to TNFα inhibitors, with higher rates of complete skin clearance and a lower tendency toward loss of response. The worst efficacy and the highest rate of loss of response were observed in the case of adalimumab. These findings support the increasing role of IL-17 and IL-23 inhibitors as preferred therapeutic options in moderate-to-severe psoriasis. Previous biologic exposure and higher BMI seem to be associated with poorer therapeutic outcomes. The most frequently observed side effect was infection, occurring mainly in patients receiving TNFα inhibitors and bimekizumab; no serious events occurred. Overall, biological treatment is an effective, rapid, and safe therapeutic option.

## Figures and Tables

**Table 1:** Basic characteristics of the analyzed group.

Number of patients	165
Sex (M/F)	90/75
Age	38.1 ± 5.66
BMI	28.9 ± 3.21
Children/adults	16/149
Alcohol abuse (Y/N)	13/152
Smoking (Y/N)	50/115
Family history of psoriasis (Y/N)	87/78
Psoriasis duration (months)	201 ± 10.6 ≈ 16.7 years
Previously treated with classic systemic agents/not treated (Y/N)	152/13
Bio-naïve/previously treated with biologics	129/36
PASI before the biological therapy	15.8 ± 0.64
DLQI before the biological therapy	18.7 ± 0.49
BSA before the biological therapy	27.6 ± 1.56
Patients with high-impact areas involvement (total)	156 (94.5%)
Patients with scalp involvement (Y/N)	136/29 (82.4%)
Patients with nail involvement (Y/N)	84/81 (50.9%)
Patients with palmoplantar involvement (Y/N)	21/144 (12.7%)
Patients with genital involvement (Y/N)	60/105 (36.3%)
Psoriatic arthritis (Y/N)	48/117 (29.1%)

Y—yes, N—no, M—male sex, F—female sex.

**Table 2:** Baseline characteristics of patients with division into particular drug classes.

Characteristic	TNF-α Inhibitors	IL-17 Inhibitors	IL-23 Inhibitors
N	30	55	80
Age, years	35.06 ± 2.19	32.98 ± 1.91	45.10 ± 1.40 **/^^^
BMI, kg/m^2^	28.16 ± 1.04	27.04 ± 0.89	29.39 ± 0.68
Sex, 0/1, n	11/22	31/29 *	55/31 *
Alcohol use, 0/1, n	32/1	57/3	75/11
Smoking, 0/1, n	24/9	41/19	58/28
Psoriasis duration, months	208.82 ± 18.00	152.57 ± 15.76	237.21 ± 17.10 ^^
Baseline PASI	15.50 ± 1.64	13.44 ± 1.11	17.66 ± 0.84 ^^
Baseline DLQI	18.21 ± 0.95	18.03 ± 0.69	19.12 ± 0.63
Baseline BSA, %	28.33 ± 3.35	21.44 ± 2.37 *	31.33 ± 2.22 ^^

*/** *means a statistically significant difference between TNF* vs. *IL-17 or IL-23 with p < 0.05/0.01; ^^/^^^ means a statistically significant difference between IL-17* vs. *IL-23 with p < 0.01/0.0001*.

**Table 3:** Basic laboratory parameters before and after 16 weeks of therapy with each class of the drugs.

Parameter	TNF (n = 30) Before	TNF (n = 30) After	IL-23 (n = 80) Before	IL-23 (n = 80) After	IL-17 (n = 55) Before	IL-17 (n = 55) After
CRP	4.18 ± 1.37	3.00 ± 0.77	5.20 ± 0.78	4.06 ± 0.79	4.37 ± 1.30	3.23 ± 0.78
WBC	7.39 ± 0.57	7.61 ± 0.41	6.95 ± 0.23	7.01 ± 0.26	6.72 ± 0.23	6.51 ± 0.24
PLT	254 ± 10.8	244 ± 13.4	236 ± 8.01	240 ± 7.76	259 ± 8.56	261 ± 8.20

## Data Availability

The raw data supporting the conclusions of this article will be made available by the authors on request. The data are not publicly available due to the protection of patient privacy.
